# Significantly enhanced biomass production of a novel bio-therapeutic strain *Lactobacillus plantarum* (AS-14) by developing low cost media cultivation strategy

**DOI:** 10.1186/s13036-017-0059-2

**Published:** 2017-05-05

**Authors:** Asma Manzoor, Javed Iqbal Qazi, Ikram ul Haq, Hamid Mukhtar, Akhtar Rasool

**Affiliations:** 1 Institute of Biochemistry and Biotechnology, University of the Punjab, Quaid-i-Azam Campus, Lahore, 54590 Pakistan; 2 Department of Zoology, University of the Punjab, Lahore, Pakistan; 3 Institute of Industrial Biotechnology, Government College University, Lahore, Pakistan; 4 Centre for Animal Sciences & Fisheries, University of Swat, Swat, Pakistan

**Keywords:** *Lactobacillus plantarum*, Cheese whey, Nitrogen sources, Response surface methodology, Medium optimization

## Abstract

**Background:**

Probiotic bacteria are becoming an important tool for improving human health, controlling diseases and enhancing immune responses. The availability of a cost effective cultivation conditions has profound effect on the efficiency and role of probiotic bacteria. Therefore the current study was conducted with an objective to develop a low cost growth medium for enhancing the biomass production of a bio-therapeutic bacterial strain *Lactobacillus plantarum* AS-14. In this work the isolation of *Lactobacillus plantarum* AS-14 bacterial strain was carried out from brinjal using cheese whey as a main carbon source. Moreover, the effect of four other nutritional factors besides cheese whey was investigated on the enhanced cell mass production by using response surface methodology (RSM).

**Results:**

The best culture medium contained 60 g/l cheese whey, 15 g/l glucose and 15 g/l corn steep liquor in addition to other minor ingredients and it resulted in maximum dry cell mass (15.41 g/l). The second-order polynomial regression model determined that the maximum cell mass production (16.02 g/l) would be obtained at temperature 40°C and pH 6.2. Comparative studies showed that cultivation using cheese whey and corn steep liquor with other components of the selected medium generated higher biomass with lower cost than that of De Man, Rogosa and Sharpe (MRS) medium under similar cultivation conditions (pH 6.2 and temperature 40°C).

**Conclusion:**

It is evident that the cell biomass of *L. Plantarum* AS-14 was enhanced by low cost cultivation conditions. Moreover, corn steep liquor and ammonium bisulphate were perceived as low-cost nitrogen sources in combination with other components to substitute yeast extract. Of all these factors, cheese whey, corn steep liquor, yeast extract and two operating conditions (temperature and pH) were found to be the most significant parameters. Thus the cost effective medium developed in this research might be used for large-scale commercial application where economics is quite likely important.

## Background

The productions of functional foods containing probiotic bacteria such as lactobacilli are gaining high significance throughout the world. These bacteria enhance the microbial safety and offer organoleptic, technological, nutritional, and health benefits to the consumers. As a consequence of the large scale production of fermented foods incorporated with probiotics, the industrial production of these bacteria at low cost is becoming more important. Therefore, growth parameters including cost, ability to produce a large number of cells, and the harvesting method should be considered while optimizing growth medium. Moreover, designing new medium for enhanced biomass production can definitely lead to more economical probiotic production [1, 2]. Among the lactobacilli, the *Lactobacillus plantarum* is the most common probiotic bacterium traditionally used in fermented foods such as, vegetables, meat and dairy products [3]. The major striking characteristic of *L. plantarum* and other lactic acid bacteria (LAB) is their ability to produce lactic acid, acetic acid and other metabolites when subjected to cultivation through batch or fed-batch fermentation.

Lactic acid bacteria are strain-dependent fastidious bacteria with respect to nutrients and environmental requirements. Rich medium and suitable conditions are the key environmental parameters required for good bacterial growth [4]. Nutrient supplements, such as yeast extract and casein hydrolysate can improve the nutritional quality of the medium as they contain growth-promoting compounds in addition to organic nitrogen and carbonaceous compounds. However, the use of these nutrient supplements in large quantities is very expensive [4]. A number of studies have shown a significantly increased lactic acid and cell biomass production in most lactobacilli in the presence of yeast extract, amino acids, protein concentrates, hydrolysates, vitamins and inorganic compounds such as (NH_4_)_2_SO_4_ and (NH_4_)_2_HPO_4_ [5–7]. Similarly, supplementing the culture medium with cheese whey (industrial waste) along with commercially available growth supplements results in enhanced cell biomass production [1, 7, 8]. However, studies regarding the use of industrial wastes (cheese whey and corn steep liquor) as a nutrient supplements for enhanced biomass yield of lactobacilli, are scarce. Cheese whey is a major by-product of dairy industry retaining 55% of milk nutrients, including lactose (4.5–5.0% w/v), soluble proteins (0.6–0.8% w/v), lipids and mineral salts [9]. Whey is normally discarded in the environment as a waste product, posing a threat to the environment due to its volume disposal and reduction in biochemical oxygen demand [10, 11]. Similarly, the corn steep liquor, a by-product of corn milling industry, has been used as an inexpensive nutrient source for fermentation. It is an excellent source of nitrogen for most microorganisms due to its high content of amino acids and polypeptides with considerable amounts of B-complex vitamins [12]. Therefore, cheese whey and corn steep liquor can be used as a low cost carbon and nitrogen sources respectively for the enhanced biomass production by *Lactobacillus*.

Increased biomass may facilitate the recovery process and reduce the production cost. Other benefits of higher amounts of biomass include shortening of the fermentation time, reduced waste water volume, and accelerated downstream processing. Besides, higher cell density of *Lactobacillus* also benefits industries to produce higher concentrations of chemicals such as lactic acid or bacteriocins. Increased biomass production of *L. plantarum* LP02 and *L. plantarum* Pi06 [13] by optimizing medium using a combination of the Taguchi array design and Box-Behnken design [14] have been recently reported. Optimizing fermentation media have been extensively studied to produce exopolysaccharides and bacteriocin [15–17] and fermentation of olive juice [18] by *L. plantarum*.

Various studies have determined LAB culture medium optimization using a time effective statistical approach, response surface methodology (RSM) [14, 18–20] instead of conventional “one factor at a time” approach. Response surface methodology with a central composite design has often been used for the optimization of biomass yield of *Lactobacillus rhamnosus* [21], *Bacillus coagulans* [22], and *Bifidobacterium longum* [23]. Nevertheless, the concomitant use of Taguchi and RSM statistical methods have not yet been employed in *Lactobacillus* culture optimization studies, while using cheese whey as a main carbon source.

Therefore, the current study was designed to determine the most significant variables among the culture parameters including cost effective carbon source cheese whey with corn steep liquor in all possible combinations for enhanced biomass production of our recently isolated and characterised *Lactobacillus plantarum* AS-I4 [24], by using Taguchi design and Box-Behnken design (RSM).

## Results and discussion

### The optimization of five nutrient variables

The three different media compositions were used for screening of preliminary dry cell mass production of *Lactobacillus plantarum* AS-14 (Table 1). Among three media compositions, medium M3 produced a higher biomass than that produced by other media except MRS. But no significant difference was observed in dry cell mass production between medium M3 and MRS under agitation condition. Five out of the ten ingredients in medium M3 were selected for medium optimization by Taguchi array design with L16 (4^5^) array (Table 4). The five independent nutrient variables included glucose (X_1_), yeast extract (*X*_2_), cheese whey (X_3_), (NH_4_)_2_SO_4_ (X_4_), and corn steep liquor (X_5_), and two operating conditions pH (A) and temperature (B), with the respective design codes of the nutrient variables and their corresponding levels, coded 1 to 4, are listed in Tables 2 and 3. All of the 16 experiments designed for media composition were conducted under static conditions. The results obtained from the optimization of 5 nutrient variables (X_1_ to X_5_) and 2 operating conditions (A, B) using Taugchi’s experimental design for the production of dry cell mass are summarized in Table 4. The highest dry cell mass production achieved in the verification experiment was 13.88 g/l as seen in run 14 (Table 4) with the X_1_ – X_5_ and A, B levels in the orderof 4, 3, 1, 4, 3, 3, and 3. First-order regression was performed by multiple regression analysis and the following regression equation Eq. 3 was obtained:

**Table 1:** Compositions of the media used for the growth of *Lactobacillus plantarum* (AS14)

Component	Media concentration (g/l)		
	M1	M2	M3
Cheese whey	-	-	75
Glucose	50	75	20
Tryptone	20	10	-
Yeast extract	8	8	8
Corn steep liquor	10	10	20
(NH_4_)_2_SO_4_	-	15	20
KH_2_PO_4_	0.2	-	0.2
Sodium acetate	-	-	5
Tween 80	-	0.2	0.2
MgSO_4_.7H_2_O	-	-	0.05
FeCl_3_	0.05	-	0.05

**Table 2:** Experimental levels of independent nutrient variable and operating conditions with their corresponding levels

	Independent Variables (g/l)	Symbol	Levels			
			1	2	3	4
Production Medium Optimization	Glucose	(X_1_)	5	10	15	20
	Yeast Extract	(*X*_2_)	2	4	6	8
	Cheese Whey	(X_3_)	30	45	60	75
	(NH_4_)_2_SO_4_	(X_4_)	5	10	15	20
	Corn steep Liquor	(X_5_)	5	10	15	20
Operating Conditions Optimization	pH (A)	(A)	6.1	6.2	6.3	6.4
	Temperature°C (B)	(B)	30	35	40	45

**Table 3:** Coded values and real values of the experimental variables for Box-Behnken method

	Independent Variables (g/l)	Symbol	Coded values		
			−1 (Low)	0 (Centre)	1 (High)
Production Medium Optimization	Glucose	(X_1_)	10	15	20
	Yeast Extract	(*X*_2_)	4	6	8
	Cheese Whey	(X_3_)	45	60	75
	(NH_4_)_2_SO_4_	(X_4_)	10	15	20
	Corn steep Liquor	(X_5_)	10	15	20
Operating Conditions Optimization	pH	(A)	6.2	6.3	6.4
	Temperature °C	(B)	35	40	45

Other composition included sodium acetate (5g/l), MgSO_4_.7H_2_O (0.3g/l), MnSO_4_.4H_2_O (0.04g/l), Tween 80 0.2

**Table 4:** Optimization of five nutrient variables (X_1_-X_5_) and two operating conditions (A, B) using Taugchi’s experimental design for the production of dry cell mass

Runs order	Nutrient variables (g/l) and operating conditions levels ^a^							Response Dry cell mass (g/l)
	X_1_	*X*_2_	X_3_	X_4_	X_5_	A	B	
1	1	2	2	2	2	1	1	8.99 ± 0.02
2	3	1	3	4	2	2	1	9.12 ± 0.06
3	2	3	4	1	2	3	1	10.22 ± 0.04
4	4	1	4	2	3	4	1	13.47 ± 0.12
5	4	4	1	3	2	1	2	13.01 ± 0.01
6	3	2	2	3	1	2	2	8.21 ± 0.07
7	4	4	2	1	4	3	2	13.55 ± 0.01
8	2	1	3	3	4	4	2	11.32 ± 0.05
9	1	3	3	3	3	2	3	10.91 ± 0.05
10	2	2	4	4	3	2	3	11.21 ± 0.07
11	2	4	2	2	1	2	3	12.87 ± 0.06
12	1	4	2	4	4	2	3	12.71 ± 0.06
13	1	4	1	1	3	3	1	11.82 ± 0.02
**14**	**4**	**3**	**1**	**4**	**3**	**3**	**3**	**13.88 ± 0.03**
15	3	3	3	2	4	4	3	11.49 ± 0.08
16	1	1	2	1	1	3	4	8.21 ± 0.05

The bold data represent that experiemental run which shows maximum L. Plantarum biomass production

^a^X_1,_ Glucose_;_ *X*_2,_ Yeast extract; X_3,_ Cheese Whey_;_ X_4,_ (NH_4_)_2_SO_4_; X_5_ corn steep liquor. Other composition included sodium acetate (5 g/l), MgSO_4_.7H_2_O (0.3 g/l), MnSO_4_.4H_2_O (0.04 g/l), Tween 80 (0.2 g/l)

3 $$ \mathrm{Dry}\kern0.5em \mathrm{cell}\kern0.5em \mathrm{mass}\kern0.5em \left(\mathrm{Ya}\right)=+13.48+0.40{\mathrm{X}}_1+0.74{\mathrm{X}}_2+0.16{\mathrm{X}}_3+7.02{\mathrm{X}}_4+0.68{\mathrm{X}}_5 $$

where Ya is the predicted response or dry cell mass; colony forming unit of *Lactobacillus plantarum* AS-14 per ml culture (CFU/ml), and X_1_, X_2_, X_3_, X_4_ and X_5_ are the coded values of the test variables glucose, yeast extract, cheese whey, (NH_4_)_2_SO_4_ and corn steep liquor, respectively. The response surface-Box-Behnken method was used to further optimize and examine the three most important factors such as glucose (X_1_), cheese whey (X_3_), and corn steep liquor (X_5_), and the results in terms of respective coded values and three levels in the medium are listed in Tables 5 and 6. The biomass reached its highest (15.41 g/l) during run 15, with nutritional levels of 15 g/l glucose, 60 g/l cheese whey, and 15 g/l corn steep liquor. A second order regression formulation was derived by ANOVA following regression as given below:

**Table 5:** Box-Behnken design of experimental response of three nutrient variables (X_1,_ glucose_;_ X_3,_ cheese Whey_;_ X_5,_ corn steep liquor) in terms of coded factor

Runs order	Nutrient variables			Response Dry cell mass (g/l)
	X_1_	*X*_2_	X_3_	
1	0	−1	0	13.66 ± 0.04
2	−1	0	1	14.96 ± 0.07
3	0	0	0	13.28 ± 0.03
4	−1	0	0	15.34 ± 0.06
5	0	1	1	15.35 ± 0.08
6	1	0	1	14.55 ± 0.01
7	−1	1	1	15.12 ± 0.11
8	0	1	−1	15.14 ± 0.16
9	−1	−1	0	15.18 ± 0.04
10	1	0	0	13.69 ± 0.06
11	1	−1	0	15.25 ± 0.07
12	1	0	−1	15.20 ± 0.06
13	0	1	−1	14.87 ± 0.05
14	0	−1	1	14.99 ± 0.08
**15**	**1**	**−1**	**1**	**15.41 ± 0.06**
16	−1	1	−1	15.17 ± 0.04

The bold data represent that experiemental run which shows maximum L. Plantarum biomass production

**Table 6:** Box-Behnken design of experimental response of two operating conditions (A, B) in terms of coded factor

Runs order	Operating conditions		Response Dry cell mass (g/l)
	A	B	
1	1	1	13.66 ± 0.08
2	0	1	14.96 ± 0.06
3	0	0	15.28 ± 0.01
4	−1	1	15.44 ± 0.04
5	−1	0	16.04 ± 0.01
6	0	1	14.55 ± 0.13
7	−1	0	16.12 ± 0.06
8	1	−1	15.14 ± 0.05
9	−1	0	15.88 ± 0.05
10	0	0	14.69 ± 0.02
11	1	0	15.55 ± 0.02
12	−1	0	16.20 ± 0.03
13	1	−1	14.87 ± 0.05
14	−1	1	15.99 ± 0.07
15	0	−1	15.91 ± 0.06
16	−1	0	16.17 ± 0.07

**Final equation in terms of coded factors:**

4 $$ \begin{array}{l}\mathrm{Dry}\kern0.5em \mathrm{cell}\kern0.5em \mathrm{mass}\kern0.5em \left(\mathrm{Ya}\right)=+12.74+2.77{\mathrm{X}}_1+1.16{\mathrm{X}}_3+9.48{\mathrm{X}}_5+0.898{\mathrm{X}}_1^2+0.0571{\mathrm{X}}_3^2+0.786{\mathrm{X}}_5^2\hbox{-} 5.34{\mathrm{X}}_1{\mathrm{X}}_3+\\ {}14.69{\mathrm{X}}_1{\mathrm{X}}_5+5.64{\mathrm{X}}_3{\mathrm{X}}_5\end{array} $$

quadratic and linear interaction effects were calculated for the optimization process, and significant coefficients were preserved to create the corresponding response surfaces. The results in the ANOVA of Table 7 showed that the independent variable (X_1_) had a significant effect, as it had a positive coefficient, according to which an increase in its concentration led to an increased yield. The independent variables X_3_ and X_5_ were also significant within the range of this study. The same is observed with the X_1_X_5_ interaction. The negative signs of the X_1_X_3_ interaction and squared variables X_1_^2^, X_3_^2^ and X_5_^2^ revealed a reduction in dry cell mass production when their concentration was increased in the system.

**Table 7:** Analysis of variance (ANOVA) for the response surface of full quadratic model for optimization of three variables (X_1,_ glucose_;_ X_3,_ Cheese Whey_;_ X_5,_ corn steep liquor)

Source	Sum of Square	Degree of freedom	Mean Square	F-value	*P*-value
X_1_	70.46	1	70.46	1182.44	0.0002
X_1_^2^	0.021	1	0.021	0.345	0.4098
X_3_	10.19	1	10.19	171.07	0.0004
X_3_^2^	1.160	1	1.160	521.321	0.001601
X_5_	34.60	1	34.60	580.57	0.00131
X_5_^2^	0.029	1	0.029	0.551	0.23481
X_1_X_3_	23.78	1	23.78	399.10	0.0001
X_1_X_5_	23.59	1	23.50	395.80	0.0035
X_3_X_5_	20.52	1	20.52	344.37	0.0003
Lack of fit	0.01336	3	0.004112	2.001	0.311310
Pure error	0.061	2	0.056		
Total	184.425	14			

R^2^: 0.9955; Adjusted R^2^:0.9936

**Fig. 1:**
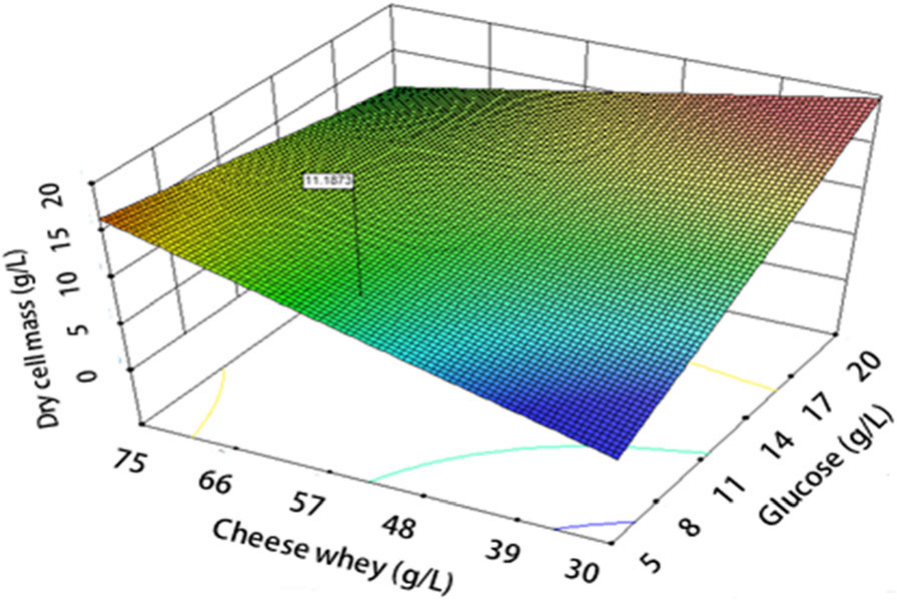
Response surface of dry cell mass production by *Lactobacillus plantarum* (AS-14), showing the interaction of glucose (X_1_) and Cheese whey (X_3_) at constant levels of corn steep liquor (15 g/l) yeast extract (5 g/l), sodium acetate (5 g/l), MgSO_4_ 7H_2_O (0.3 g/l), and MnSO_4_ 4H_2_O (0.04 g/l)

**Fig. 2:**
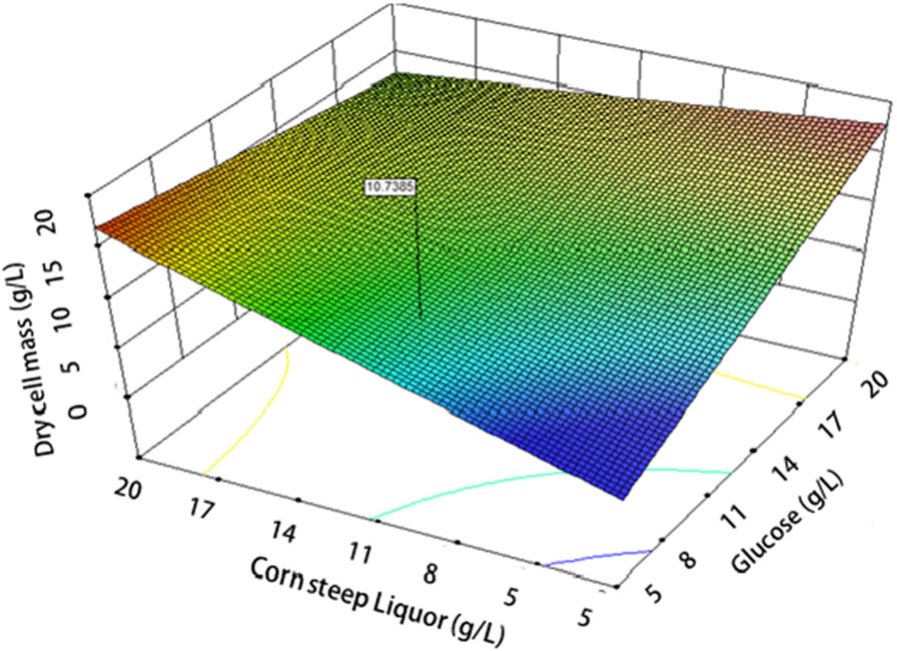
Response surface of dry cell mass production by *Lactobacillus plantarum* (AS-14), showing the interaction of glucose (X_1_) and corn steep liquor (X_5_) at constant levels of cheese whey (60 g/l) yeast extract (5 g/l), Sodium acetate (5 g/l), MgSO_4_ 7H_2_O (0.3 g/l), and MnSO_4_ 4H_2_O (0.04 g/l)

**Fig. 3:**
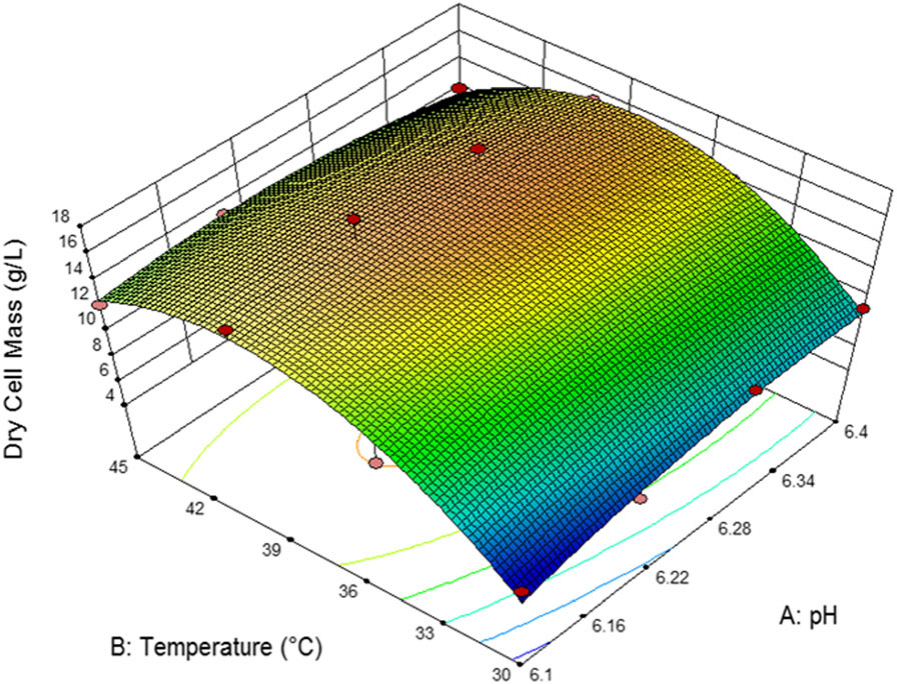
Response surface plot in relation to temperature and pH for dry mass production

**Fig. 4:**
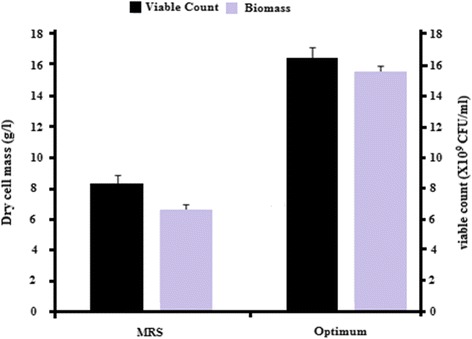
Comparison of dry cell mass and viable counts of *Lactobacillus plantarum* (AS-14) cultivated in MS medium and the optimum medium. Fermentation conditions: 5% inoculum, 40 °C, 24 h, at 100 ml medium/250 ml Hinton flask without shaking. Each value represents the mean columns are significantly different (*p* < 0.05) compared to MRS medium

It is shown that a higher concentration of cheese whey resulted in higher biomass production (Figs. 1, 2 and 3). However, a higher glucose and corn steep liquor concentrations might have resulted in inhibited cell growth. The lack of fit of the model was observed non-significant that showed good correlation between the data and the model. The high R^2^ value (0.9936) indicated that the data were close to the predicted values from the model. An optimized formulation of nutrition levels was suggested from the software at the following concentrations: 15 g/l glucose, 60 g/l cheese whey, and 15 g/l corn steep liquor. Live cells were also counted for comparison and to assure viability of cells in the MRS and optimized culture media (>1 × 10^10^ CFU/ml), (Fig. 4). The results indicated that both biomass and viable counts in the optimized culture medium were significantly higher than those in the MRS medium. Because the enhanced biomass was almost in proportion to the increase in viable cell numbers, it was deduced that volumes (or sizes) per cell were similar in each culture and that the cells in the optimum culture were alive.

### The optimization of physical operating conditions

Tables 4 and 6 show the design matrix of the variables in coded units with the experimental results. The highest dry cell mass production was 16.20 g/l as seen in run 12. For the experimental data, following equation was obtained after multiple regression analysis:

5 $$ \mathrm{Y}\mathrm{b}={\mathrm{b}}_0+{\mathrm{b}}_1\mathrm{A}+{\mathrm{b}}_2\mathrm{B}+{\mathrm{b}}_{12}\mathrm{AB}+{\mathrm{b}}_{11}{\mathrm{A}}^2+{\mathrm{b}}_{22}{\mathrm{B}}^2 $$

$$ \mathrm{Y}\mathrm{b}=16.04+0.61\mathrm{A}+2.54\mathrm{B}\hbox{-} 1.07\mathrm{AB}\hbox{-} 1.09{\mathrm{A}}^2\hbox{-} 5.36{\mathrm{B}}^2 $$

where Yb is the predicted response, i.e. dry cell mass g/l, A and B are the coded values of the test variables temperature and pH, respectively. Table 8 shows the results of the second-order response surface models for dry cell mass production in the form of analysis of variance (ANOVA). The ANOVA of the quadratic regression model demonstrates that the model is very significant as evident from very low probability value [*p*-value Prob > F < 0.0001]. The R-squared of 0.9411 is in reasonable agreement with the “adj R-squared” of 0.9083; i.e. the difference is less than 0.1. The coefficientof determination (R^2^) indicating, that 94.11% of the variability in the response could be explainedby the model. The adjusted determination coefficient (adj. R^2^ = 0.9083) was also satisfactory for confirming the significance of the model. Figure 3 displays the surface response plot of the model equation. In Eq. 3, only the isolated variable A and variable B significantly influence the process. A positive but lower value of A and higher value of B demonstrate that a rise in temperature and reduction in pH lead to an increased dry cell mass production. The determination coefficient was 0.9083, indicating that 90.9% of the variability in the response could be explained by the model. The coordinates of the stationary points for dry cell mass production were calculated from the complete Eq. 3. Figure 3 illustrates that an increase in temperature leads to increased production of dry cell mass. Maximal dry cell mass production was obtained at pH 6.2. The optimal range for dry cell mass production was from 34 to 39.6°C and pH = 6.1 to 6.4.

**Table 8:** Analysis of variance (ANOVA) for the response surface of full quadratic model for optimization of two variables (A_,_ pH_;_ B, Temperature)

Source	Sum of Square	Degree of freedom	Mean Square	F-value	*P*-value
A	43.30	1	43.30	38.01	0.0021
B	56.28	1	56.28	51.34	0.0001
AB	5.65	1	5.65	5.16	0.0493
A2	3.45	1	3.45	3.15	0.1096
B2	84.01	1	84.01	76.64	<0.0001
Lack of fit	0.0125	3	0.00291	1.809	0.37651
Pure error	0.041	2	0.096		
Total	192.75	10			

R^2^: 0.9411 Adjusted R^2^: 0.9083

Statistical design of experiments can be employed to model the relationship between certain variables and one or more responses in process. Since the cost of culture medium has a remarkable impact on the mass production of probiotics, the optimization of growth conditions, substitution with low-price nutrient ingredients and simplification of medium are vital for their economical production. Taguchi method can be employed to screen the significant nutritional parameters and design a simple medium. Response surface methodology can be employed to estimate a polynomial model representing the effect of significant factors on viable cell counts in the probiotic products, as well as to optimize the process variables. Such combination of statistical design of experiments would be useful to optimize bioprocesses. Many workers employed a two-phase procedure including a screening phase through Taguchi and an optimization phase through Box-Behnken design to develop an economical broth for the growth of Lactobacilli for example *L. casei* ATCC 334 [2], *Lactobacillus* sp. LMI8 [10], *Lactobacillus plantarum* Pi06 [13].

The selected lactobacilli strains were growing relatively slower in the MRS broth. The growth medium was therefore, reformulated and simplifiedby using three different media compositions. Furthermore, the extensive use of nitrogenous sources such as peptones from poultry or beef extract has been environment un-friendly due to high amount of waste. Therefore, we used cheese whey as a low-cost carbon source, with corn steep liquor and ammonium sulphate as low cost nitrogen sources. A step towards simplification is the omission of peptone and the evaluation of the remaining components on the growth performance of the strain. Each component was tested in arange of different concentrations, while all the remaining parameters were kept constant, equal to the initial concentration of the MRS medium. According to our results, there was no significant difference in dry cell mass production between optimum medium M3 and MRS under agitation. Generally, under non agitating conditions, higher dry cell mass was acquired as compared to shaking. These results indicate that *L. plantarum* strain AS-14 is an anaerobic microorganism. With Taguchi array design by factor optimization, the initial medium opted for further optimization experiments was medium M3.

The growth results on MRS medium reported by Zacharof and Lovitt [25] showed that this medium was unsuitable for intensive propagation of *Lactobacillus plantarum* NCIMB 8014, because medium containing numerous nitrogen sources did not facilitate good growth. The absence of peptone from the medium lead to an improved growth rate and higher growth yields of the bacteria, therefore yeast extract was chosen as a primary and sole source of nitrogen.

Several studies [26–29] have introduced the idea of partial dependence of biomass development and metabolites production by lactobacilli on the amount of nitrogen sources like yeast extract in defined growth media. Yeast extract serves as the carbon, nitrogen and vitamin source needed to satisfy the growth requirements of the microorganisms. Several studies have underlined the important influence of metal ions on the growth of lactobacilli [29–31]. We have also evaluated the effect of yeast extract, ammonium bi-sulphate, manganese and magnesium salts concentrations on dry cell mass production but according to results, they are necessary for growth but have no significant effect on response surface of growth. As the yield of biomass in all anaerobic bacteria was strongly depends on carbohydrate feed [27], glucose was used as energy source.

The 3D response surface plots are graphical representations of the regression equation; they were plotted to show the interaction of the variables and to identify the optimum level of each variable for a maximum response (Figs. 1, 2 and 3). Each response surface for biomass production represented different combinations of two test variables at once. As a result, glucose (X1) and dominant nutrients cheese whey (X3) and corn steep liquor (X5) significantly enhanced the biomass production while, yeast extract did not significantly affect the cell growth. Moreover, it was shown that the log value of viable cells in the optimized medium based on cheese whey and corn steep liquor was increased as compare to complex and expensive MRS medium (Fig. 4). Further, cheese whey and corn steep liquor as a low cost carbon and nitrogen sources could be an appropriate substitute for many other carbon and nitrogen sources such as glucose, yeast extract, KH_2_PO_4_ in different medium compositions.

Combining all the optimised growth parameters in the desired quantities formulated a liquid medium. This modified medium served the aim of enhancing the cellular productivity, ensuring high growth. When comparing the growth of the selected lactobacilli on MRS and the formulated media (Fig. 4), it was clearly shown that the log value of viable cells in the optimized medium based on cheese whey and corn steep liquor was increased to that in the complex and expensive MRS medium which demonstrated that the optimized medium improves growth with a significant increase in dry cell mass.

Corn steep liquor and lactose have long been proven inexpensive dominant nutrients, alternative to much more expensive materials, such as yeast extract and peptone. It has been used as an alternative nitrogen and carbon sources for optimizing lactic acid production by LAB [21, 32]. Because LAB are nutritionally fastidious and require various amino acids and vitamins for growth, choosing a suitable nitrogen source appears to be very important. Lactose and corn steep liquor are the dominant nutrients that control the biosynthesis of lactic acid produced by LAB. Hence, a strong interaction between them for lactic acid fermentation is inevitable. Lima et al. [10] proposed that maximal lactic acid production (18.31 g/l) was obtained for values of cheese whey (total lactose 55 g/l) and corn steep liquor (15 g/l) in the central point region.

Hwang et al. [13] investigated the fermentative production of dry cell mass of *Lactobacillus plantarum* Pi06, to 8.94 g/l from three response variables of glucose, yeast extract, and corn steep liquor. The resulting optimum medium consisted of 35 g/l glucose, 35 g/l yeast extract, and 40 ml/lcorn steep liquor by using Box-Behnken method. Wee et al. [33] explored the lactic acid production (up to 91 g/l) using *Lactobacillus* sp. RKY1 from corn steep liquor (15–60 g/l) and cheese whey containing 100 g/l of lactose as cheap raw materials. Plessas et al. [34] used cheese whey (initial lactose concentrationof 36 g/l) and sour dough (1%), which resulted in maximum production of 6.9 g/l of lactic acid (by single culture) and 8.8 g/l of lactic acid (by mixed culture).

To further optimize the growth rate and the dry cell mass concentration, the effect of physical operating conditions, temperature and pH over the growth of Lactobacilli was investigated in 3 l fermenter containing the optimum formulated medium. The optimum temperature and pH resulted in 16.20 g/l dry cell mass was 40°C and 6.2, respectively. At the stationary point, in the MRS broth (pH 6.0), the dry cell mass concentration was 8.94 g/l, while in the medium without pH control, it was only 6.02 g/l. These values represent an increase of 165% of dry cell mass production when the pH of the supplemented hydrolysate was controlled.

An incubation temperature of lactobacilli in the range of 25 to 38°C was proposed by several researchers [26–29]. Zacharof and Lovitt [25] reported that the maximum specific growth rate of three Lactobacilli was enhanced at controlled pH6.5, though in the cases of *L. Lactis* and *L. Plantarum* pH 7 also supported good growth. These experiments gave higher biomass yields and maximum specific growth rates as compared to the uncontrolled pH growth systems.

## Conclusion

The *Lactobacillus plantarum* AS-14 proves to have great potential for dry cell mass production in the presence of cheese whey and corn steep liquor. Moreover, MRS medium, although it can support the growth of lactobacilli, is unsuitable for use in large quantities owing to its high formulation cost and potential environmental hazards. Hence this study would be a great asset to address the limitations caused by conventional MRS medium for the growth of *Lactobacillus plantarum*. Hence we present a simplified, cost-effective medium that has the potential to be employed in the industrial production of different types of Lactobacilli used in dairy products.

## Methods

### Microorganism

Recently, we have isolated and characterised fifty four different species from spoiled fruits and vegetables [24] and among these species novel strain of *Lactobacillus plantarum* AS-14 was used for current study. The AS-14 strain was isolated and characterised for the first time from rotten vegetables (brinjal) collected from local fruit market of Sargodha, Punjab Pakistan. The strain was stored in de Man, Rogosa and Sharpe (MRS) broth with 20% glycerol at −20°C.

### Media composition and growth conditions

Whey powder containing 82% lactose was obtained from (Noorpur Dairy Industry, Bhalwal Sargodha). Deproteinization of whey was carried out by heat treatment (100°C for 15 min) of the acidified (pH 4.0) whey solution with some modification in the method reported by Lima et al. [10]. The resulting solution was centrifuged at 12,000X *g* and the supernatant was diluted to reach the desired lactose concentration. Corn steep liquor was obtained from Refhan Industries Pvt. Ltd., Lahore, Pakistan.

Inoculum of *L. Plantarum* was prepared by transferring glycerol stock culture (1ml) to an Erlenmeyer flask containing 50 ml of MRS medium and incubated at 37°C for 18 h (time required for the microorganism to reach the exponential growth phase) without agitation. Erlenmeyer flasks containing the production medium were inoculated with 1% (V/V) inoculum grown in the MRS medium. The composition of MRS medium was (g/l): peptone 10, yeast extract 5, beef extract 10, glucose 20, sodium acetate 5, Na_2_HPO_4_ · 2H_2_O 2, triammonium citrate 2, MgSO_4_ · 7H_2_O 0.1 and MnSO_4_ · 4H_2_O 0.05. For the optimization of dry cell mass (DCM) production, the experiments were performed in 250 ml Erlenmeyer flasks containing 100 ml of production medium. Three different media compositions were screened to identify a suitable one for further experiments (Table 1). All media were adjusted to pH 6.2 before sterilization at 121°C for 15 min. The culture with the highest biomass production was selected for subsequent optimization experiments.

The production medium consisted of the same salts used in the growth medium, with the addition of whey lactose (30 to 75 g/l), corn steep liquor (5 to 20 g/l) and (NH_4_)_2_SO_4_ (5 to 20 g/l). Initial pH was 6.5 and it was not kept constant throughout the experiments. The optimization of temperature (30°C to 45°C) and pH (6.1 to 6.4) was also carried out in a 3.0 l fermenter (Eyela Tokyo. Jar Fermenter MBF) with working volume of 1.5 l.

### Measurement of dry cell mass

The dry cell mass of the fermented broth was measured by a UV-visible spectrophotometer (Shimadzu Co, Tokyo, Japan). Aliquots of the cell culture obtained at different time intervals were centrifuged, washed twice and suspended in distilled water and their absorbance was measured at 600 nm. Washed cells were dried in an oven at 80°C for 16–24 h and weighed to constant weight. Dry cell mass was determined by a calibration curve measured at the absorbance values of cell density (OD_600nm_) and dry cell weight (g/l). The glucose concentration in the supernatant was measured by the dinitrosalicylic acid (DNS) method. The number of viable cells of *L.plantarum* AS-14 was determined using serial 10-fold dilution in sterile physiological saline. Secondary dilutions (0.1 ml) from 10^4^ to 10^8^ were injected into anaerobic tubes (containing MRS agar, 50°C) and immediately rotated in ice water. Anaerobic tubes (capped with butyl rubber stoppers) were placed in an incubator at 37°C for 24–48 h, and the colony-forming units were estimated by counting viable cells cultivated in MRS agar plates at 37°C after a series of sample dilutions in 0.85% physiological saline.

### Optimization of growth media

The response surface methodology was conducted by applying Taguchi array design to understand the interaction of various variables and operating conditions, used to find the optimum concentration of the main medium components that affect the response (dry cell mass). To examine the effect of five growth factors (X_1_ to X_5_) on the production of dry cell mass, standard orthogonal L_16_ (4^5^) factorial arrays design was used for designing the experiment. The L_16_ represents the Latin square and the number of experimental runs, respectively. Every run set consisted of a particular combination of levels and factors. The value of each level is listed in Table 2. Each selected combination of factors and levels was tested in a Hinton flask containing 100 ml of culture under static conditions. To estimate the optimal point of four important variables from the results of the Taguchi array design, a second order polynomial function, the Box-Behnken Design (BBD) method was fitted to the experimental results. Thus, the influence of all experimental variables, factors and interaction effects on the response was investigated. The objective of the second experiment was to obtain a more precise estimate of the optimal operating conditions for the factors involved. Thus, ABBD factorial 3^3^ experimental design was developed with four variables at three levels. Only a small number of experimental runs (i.e. 16 runs) were necessary for the optimization of nutrition variables (cheese whey, glucose and yeast extract) and with two variables (temperature and pH) listed in Table 3.

1 $$ \begin{array}{l}\mathrm{Ya}={\mathrm{a}}_0+{\mathrm{a}}_1{\mathrm{X}}_1+{\mathrm{a}}_2{\mathrm{X}}_2+{\mathrm{a}}_3{\mathrm{X}}_3+{\mathrm{a}}_4{\mathrm{X}}_4+{\mathrm{a}}_5{\mathrm{X}}_5+{\mathrm{a}}_{12}{\mathrm{X}}_1{\mathrm{X}}_2+{\mathrm{a}}_{13}{\mathrm{X}}_1{\mathrm{X}}_3+{\mathrm{a}}_{14}{\mathrm{X}}_1{\mathrm{X}}_4+{\mathrm{a}}_{15}{\mathrm{X}}_1{\mathrm{X}}_5\\ {}{\mathrm{a}}_{23}{\mathrm{X}}_2{\mathrm{X}}_3+{\mathrm{a}}_{11}{\mathrm{X}}_1^2+{\mathrm{a}}_{22}{\mathrm{X}}_2^2+{\mathrm{a}}_{33}{\mathrm{X}}_3^2+{\mathrm{a}}_{44}{\mathrm{X}}_4^2+{\mathrm{a}}_{55}{\mathrm{X}}_5^2\end{array} $$

2 $$ \mathrm{Y}\mathrm{b}={\mathrm{b}}_0+{\mathrm{b}}_1\mathrm{A}+{\mathrm{b}}_2\mathrm{B}+{\mathrm{b}}_{12}\mathrm{AB}+{\mathrm{b}}_{11}{\mathrm{A}}^2+{\mathrm{b}}_{22}{\mathrm{B}}^2 $$

where Ya and Yb are the predicted response (dry cell biomass) values, a_0,_ b_0_ are the constants, a_1_, b_1,_ a_2,_ b_2,_ a_3,_ a_4_ and a_5_ are the linear coefficients; a_12,_ b_12,,_ a_14,_ a_15_ and a_23,_ are the cross product coefficients; and a_11,_ b_11,_ a_22,_ b_22,_ a_33,_ a_44,_ a_55_ are the quadratic coefficients.

### Data analysis

The effects of the factors on surface tension for biomass production were statistically analysed with analysis of variance (ANOVA). Response surface methodology was carried out using Design Expert software package (version 9.0.3.1, Stat-Ease Inc., USA).

## Abbreviation

BBD: Box behnken design
DCM: Dry cell mass
DNS: Dinitrosalicyclic acid
*L. Lactis*: *Lactoccocus lactis*
MRS: de Man, Rogosa and Sharpe
RSM: Response surface methodology
